# 3D Printing of an Oil/Water Mixture Separator with In Situ Demulsification and Separation

**DOI:** 10.3390/polym11050774

**Published:** 2019-05-01

**Authors:** Changyou Yan, Shuanhong Ma, Zhongying Ji, Yuxiong Guo, Zhilu Liu, Xiaoqin Zhang, Xiaolong Wang

**Affiliations:** 1 State Key Laboratory of Solid Lubrication, Lanzhou Institute of Chemical Physics, Chinese Academy of Sciences, Lanzhou 730000, China; yanchangyou@licp.cas.cn (C.Y.); mashuanhong@licp.cas.cn (S.M.); zyjilicp@163.com (Z.J.); guoyuxiong91@163.com (Y.G.); zlliu@licp.cas.cn (Z.L.); 2 Center of Materials Science and Optoelectronics Engineering, University of Chinese Academy of Sciences, Beijing 100039, China; 3 Yiwu R&D Centre for Functional Materials, LICP, CAS, Yiwu 322000, China; 4 School of Chemistry and Chemical Engineering, Shihezi University, Shihezi 832003, China

**Keywords:** 3D printing, superhydrophilic, underwater superoleophobic, oil/water separation, in situ demulsification and separation, oil skimmer

## Abstract

Currently, many meshes, membranes, and fabrics with extreme wettability of superhydrophobicity/superoleophilicity, or superhydrophilicity and underwater superoleophobicity are promising candidates for oil/water mixture separation. Nevertheless, a facile yet effective way to design and fabricate porous mesh still remains challenging. In this work, fused deposition modeling (FDM) 3D printing of Fe/polylactic acid (PLA) composites was employed to fabricate superhydrophilic and underwater superoleophobic mesh (S-USM) with hydrogel coatings via the surface polymerization of Fe(II)-mediated redox reaction. In addition, salt of aluminum chloride was incorporated within the hydrogel coating, which was attributed to strengthening the demulsification of oil-in-water emulsions, resulting in efficient separation of oil-in-water mixtures. The S-USM was efficient for a wide range of oil-in-water mixtures, such as dodecane, diesel, vegetable oil, and even crude oil, with a separation efficiency of up to 85%. In this study, the flexible design and fabrication of 3D printing were used for the facile creation of spherical oil skimmers with hydrogel coatings that were capable of removing the floating oil. Most importantly, this work is expected to promote post-treatment processes using 3D printing as a new manufacturing technology and, in this way, a series of devices of specific shape and function will be expanded to satisfy desired requirements and bring great convenience to personal life.

## 1. Introduction

Despite the recent emergence and increasing practical feasibility of conventional techniques involving oil skimmers, centrifuges, coalescers, and flotation technologies, the separation of oil/water mixtures cannot be easily handled when faced with wastewater from metal workshops, textiles, leather, and petrochemicals, as well as frequent oil spill accidents [1,2]. What makes oil/water mixtures difficult to separate is that oil/water mixtures often contain immiscible mixtures and emulsified mixtures in which micro oil/water droplets are under a stable state [3,4,5,6,7]. Herein, the key to achieving a high separation efficiency for emulsified oil-in-water mixtures is to break stable oil-in-water emulsions.

Currently, extreme wetting materials, such as superhydrophobic/superoleophilic or superhydrophilic and underwater superoleophobic filters [8,9], oil absorptions [10,11,12,13,14,15], and membranes [16,17,18,19], are promising candidates for separating oil-in-water mixtures. Among these, membranes are deemed to be highly efficient and economical [20]. Recent progress in the fabrication of highly porous membranes has been enabled by textured metal meshes [21], assembled nanostructures [22], as well as electrospinning [23,24,25,26]. However, membranes with complicated geometries and diversified composites are still difficult to fabricate.

Allowing flexible design and freeform in three dimensions, 3D printing is a promising method to construct structurally functional devices with post-chemical modifications, and has drawn intense interest regarding application in tissue engineering [27,28,29], microfluidics [30,31], and special wetting surfaces [32,33,34]. Hydrogel, a crosslinked network full of water, is an example of typical hydrophilic materials. Owing to their excellent capabilities for absorbing and holding water, hydrogels are promising candidates to be used to increase wettability. The Taubert research group [35] deposited a hydrogel/calcium phosphate hybrid layer on 3D printed poly(lactic acid) scaffolds for biomaterial fabrication. The Irvine research group [36] further studied the biomaterial surface made by hydrogels on 3D printed substrates for bioartificial blood vessels. The Bashir research group [37] demonstrated separation of orthogonal functions enabled by 3D printing within a hydrogel particle. The Jiang research group reported using hydrogel-coated mesh for oil/water separation [38,39]. Furthermore, polyacrylamide (PAM) hydrogel has been broadly researched for wastewater treatment as a coagulator or flocculator [40,41,42]. Hence, the combination of 3D printing mesh and PAM hydrogel is promising for oil/water separation.

**Scheme 1:**
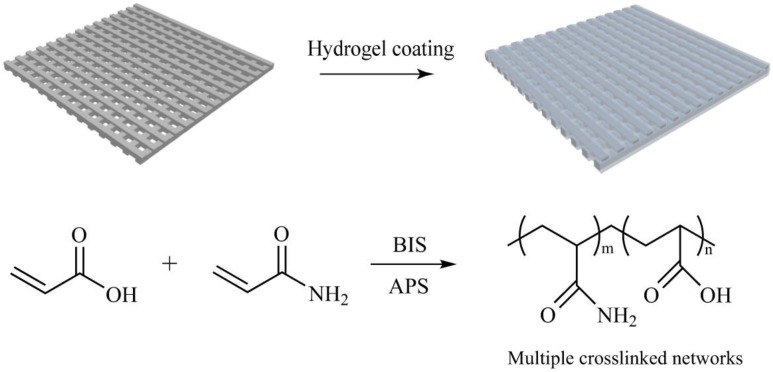
Illustration for the fabrication of S-USM. The hydrogel coating process involves polymerization of AA and AM, in which *N*,*N*′-methylenebisacrylamide (BIS) serves as a crosslinker and ammonium persulfate (APS) acts as a catalyst.

In this work, taking advantage of inherent superhydrophilicity and underwater superoleophobicity of hydrogel, we prepared meshes used for oil/water separation by combining 3D printing with hydrogel-coated modification (Scheme 1). Specially, Fe/PLA composites were extruded into filament and then printed into orthogonal meshes using a fused deposition modeling (FDM) 3D printer. The printed mesh was suspended in acrylic acid (AA) and acrylamide (AM) solution to induce PAA/AM hydrogel coating, bounding, and growing on mesh via the surface polymerization of Fe(II)-mediated redox reaction [43,44]. After being immersed in inorganic salt solution, the inorganic salt was incorporated into hydrogel coating to strengthen demulsification of oil-in-water emulsions. Due to superhydrophilic and underwater superoleophobic properties of hydrogel coating, the underwater oil contact angle of superhydrophilic and underwater superoleophobic mesh (S-USM) was over 150 °C with a low adhesion force. The salt-containing S-USM acted as a selective separation membrane, which allowed water to permeate while repelling oil droplets. In order to investigate the separation capability of salt-containing S-USM, four representative kinds of oil mixtures, namely dodecane-in-water, diesel-in-water, vegetable oil-in-water, and crude oil-in-water, were chosen and examined with simple homemade equipment, which demonstrated that S-USM could separate oil/water mixtures with a separation efficiency up to 85%.

## 2. Materials and Methods

### 2.1. Materials

Polylactic acid (PLA, melting temperature: 200–220 °C) was available as an industrial product (UNIC Technology (Suzhou) Ltd., Suzhou, China). Fe powders were obtained from Nakano Shinsho. Acrylic acid (AA, TCI, Tokyo, Japan), acrylamide (AM, Sigma-Aldrich, St. Louis, MO, USA), crosslinker *N,N*′-methylenebisacrylamide (BIS, Sinopharm Chemical Reagent Co. Ltd., Shanghai, China), ammonium persulfate (APS, Tianjin Chemical Reagents Corp., Tianjin, China), polyacrylamide (PAM, Aldrich) were used as received. Sodium chloride (NaCl), calcium chloride (CaCl_2_), and aluminum chloride (AlCl_3_) were purchased from Sinopharm Group Chemical Reagent Co. Ltd., Beijing, China without any treatment before use. The distilled water used in the experiment was made in the laboratory. Other chemical reagents were of analytical grade and used as received in this work.

### 2.2. Preparation of Fe/PLA Composites Filament

To prepare the Fe/PLA composite filament, the PLA particles were first dissolved in CHCl_3_ solvent along with magnetic stirring in a beaker for 24 h at room temperature to obtain pre-PLA solution. After the PLA was completely dissolved, Fe powders were added into pre-PLA solution with mechanical agitation at 500 rpm for 10 min to allow Fe powders to disperse homogeneously. Then, the Fe/PLA mixture was transferred to a platform inside a fume hood to evaporate CHCl_3_ solvent and remove residual bubbles. Following this, the Fe/PLA mixture was dried, cut into small cubic grains of size less than 2 (length) × 2 (width) × 2 (thickness) mm^3^, and fabricated into filaments of 1.75 mm diameter using a plastic extrusion processing machine (SJZS-10B, Ruiming, Wuhan, China) for FDM printing. In detail, Fe/PLA composites were gradually melted at 210 °C and squeezed out from an extrusion processing machine with the aid of drag. In the above steps, the weight ratio of Fe/PLA was 10/30.

### 2.3. Printing Meshes

Printing was carried out utilizing a commercially available FDM 3D printer (Hori X500D, China) with a nozzle of 400 μm in diameter. The Fe/PLA composite filament was heated to 210 °C and squeezed out. With the platform’s motion along a single axis, the extruded filaments were stacked in parallel rows, and a second floor was then built up in an orthogonal direction. In this case, the platform speed was 20 mm/s with an extrusion speed of 25 mm/s. Due to the orthogonal structure of the mesh, all pores were rectangular in shape. The size of the pores was calculated according to

S = L − d,(1)

using the spacing distance (L) and the diameter of a single stick (d). With the aid of flexible designing and 3D printing, it was convenient to acquire a variety of pore scales from 100% to 200%.

### 2.4. Fabrication of Salt-Containing S-USM

Two steps were needed to obtain salt-containing S-USM. Firstly, the printed mesh was immersed into AA/AM solution for hydrogel coating. The deionized water (20 mL), AM (4.26 g, 0.06 mol), AA (0.436 g, 0.006 mol), chemical crosslinker BIS (0.01 g, 4.38 × 10^−5^ mol), and initiator APS (0.01 g, 6.48 × 10^−5^ mol) were mixed under stirring in a flask with nitrogen blowing. Then the printed mesh was immersed in the solution. The time for which the mesh was suspended in hydrogel solution, in the range of 15 s to 90 s, was dependent on the thickness of the hydrogel layer. After the hydrogel layer polymerization finished, the mesh was taken out and washed with deionized water to remove excess monomer. Secondly, the hydrogel-coated mesh was immersed in saturated salt AlCl_3_ solution. Then the salt-containing S-USM was obtained.

### 2.5. Separation Performance of Salt-Containing S-USM

The equipment for oil/water separation experiments was homemade, whereby a mesh was fixed between two glass tubes. The oil/water mixtures were poured onto the mesh and separated by gravity. In this experiment, four representative kinds of oil mixtures (dodecane-in-water, diesel-in-water, vegetable oil-in-water, and crude oil-in-water) were adopted to test the separation efficiency of salt-containing S-USM. After separation, the separation efficiency was calculated using the weight ratio of the collected water and original water. The droplet size of oil/water mixtures before and after separation was detected by optical microscope and dynamic light scattering. In addition, further characterization for separation processes were observed in an optical microscope. The recycle test of separation was carried out using several repeated experiments. Furthermore, the mesh was also designed into a particular spherical skimmer that succeeded in collecting floating oil.

### 2.6. Characterizations

The morphologies of the S-USM were analyzed utilizing a field emission scanning electron microscope (JSM-6700F, JEOL, Tokyo, Japan) and optical microscope (Olympus BX51, Tokyo, Japan). The contact angles in air and underwater oil droplets were measured via a contact angle tester (ThetaLite, Biolin Scientific, Manchester, UK) in sessile mode at ambient temperature. The adhesion forces between oil and S-USM were calculated using a high-sensitivity dynamic contact angle analyzer (Data-Physics DCAT11, Filderstadt, Germany). The volume of oil droplets used in contact angle measurement was 10 μL, while the approaching and retracting speed of the droplets to the substrate was 0.05 mm/s.

## 3. Results and Discussion

### 3.1. Morphologies of 3D-Printed Mesh

**Figure 1:**
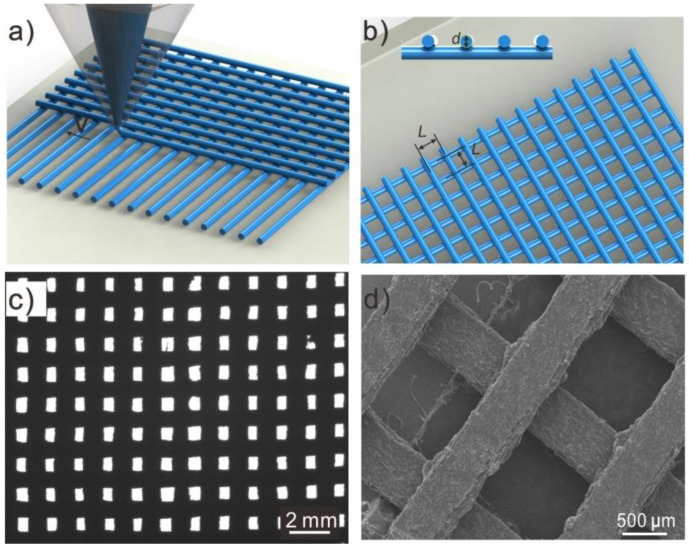
(**a**) Typical schematic illustration of the fused deposition modeling (FDM) 3D printing process. (**b**) Design of 3D printing orthogonal mesh with parameter of diameter, layers, and spacing. (**c**,**d**) Optical and SEM photographs of Fe/PLA composites mesh with through hole.

The printing process of mesh is shown in Figure 1a. The mesh was fabricated in two orthogonal layers via FDM 3D printing of Fe/PLA composite filaments (Figure S1). The filaments were extruded using heat extrusion (210 °C) and printed in a layer-by-layer sequence., By controlling the printing parameters shown in Figure 1b, such as stick diameter (d), spacing distance (L), and motion speed (V), a series of meshes with different spacing widths was formed (Figure S2). In addition, the advantage of FDM 3D printing in feasible designing and free forming was obvious, especially for preparing in the macro millimeter to large scale. The lateral dimensions were kept at 20 mm × 20 mm in Figure 1c, with a facile to larger size, if needed. In the SEM image (Figure 1d), 3D-printed mesh is fixed using two orthogonal layers and without collapsing, which is important for offering support to the growing hydrogel coating.

### 3.2. Fabrication of Hydrogel Coating

**Figure 2:**
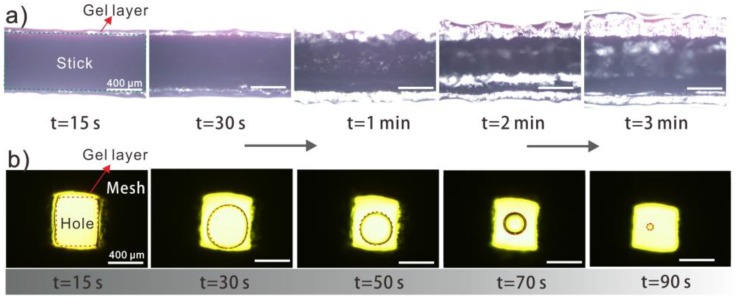
(**a**) The optical microscope photos of hydrogel coating growth on single Fe/PLA stick with reaction time. (**b**) The observation of hydrogel thickness increasing from approximately 50 to 400 μm until the pore is almost blocked, showing feasibility in controlling pore size.

Hydrogel coating was prepared via the surface polymerization in an Fe (II)-mediated redox reaction during the process of suspending printed Fe/PLA mesh in AA/AM solution. This one-step method of hydrogel coating on Fe/PLA composite mesh was facile and rapid. The optical images in Figure 2 show the process of the hydrogel coating thickness increasing. In Figure 2a, a single stick was wrapped by hydrogel coating. It is noted that the initial thickness of the hydrogel layer was smooth and uniform at 15 s, but corrugated when extending polymerization time, especially after 2 min. The maximum thickness of hydrogel coating is approximately 400 μm, resulting in a tight hydrogel layer enclosing over the stick (Figure S3a,b), whereas for an orthogonal mesh (Figure 2b), holes surrounded by hydrogel walls were generated, and with the increase of hydrogel wall thickness, the diameter of the holes decreased [43]. Through energy-dispersive spectrometry (EDS) in Figure S3d–f, it is obvious that the C, N, and O elements are distributed on the entire S-USM surface, with the atomic percent of C, N, and O being 43.8%, 29.2%, and 28.0%, respectively.

### 3.3. Wettability of S-USM

**Figure 3:**
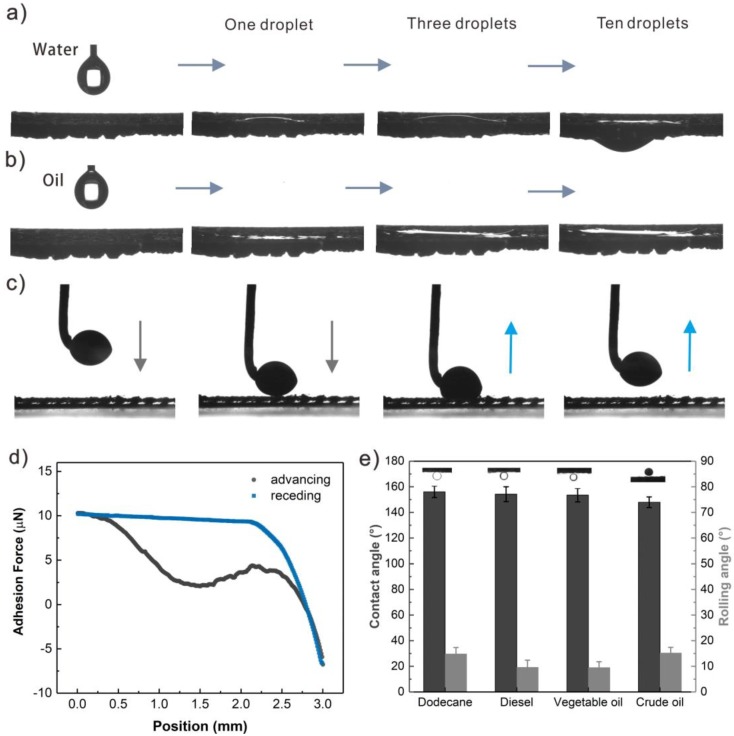
Wettability of S-USM. (**a**) Water contact angle of S-USM, where water droplets spread and permeate through the S-USM, whereas (**b**) oil droplets only spread on the mesh. (**c**,**d**) Crude oil droplets are repulsed over the S-USM underwater with a low adhesion force. (**e**) The underwater contact angle and sliding angle of four kinds of oil (dodecane, diesel, vegetable oil, and crude oil).

As is well known, the membrane separation capacity of oil/water mixtures depends largely on membrane wettability [2]. In order to investigate the wetting properties of S-USM, we characterized the contact angles and permeating behavior of multi-drops of water or oil, with results in Figure 3a,b. In air, the water droplets infiltrated the S-USM rapidly with a contact angle of close to zero (Figure 3a). While oil droplets also spread on the S-USM at a low contact angle of less 10°, they did not permeate through the S-USM, even when more than ten droplets were added onto the mesh (Figure 3b). Similarly, in the process of oil/water separation, water droplets would meet with the S-USM, and permeated through the mesh without hesitation, as well as the residue of oil droplets being blocked above the mesh. This process often takes place in a water-filled environment in most cases of oil/water mixture separation, due to the differentiation density of oil and water. Furthermore, dynamic underwater oil (crude oil) contact was observed, as shown in Figure 3c, as well as adhesion force (Figure 3d). In the process of oil receding, no residual oil droplets were visible on S-USM with an underwater oil adhesion force less than 1 μN. Four kinds of underwater oil contact angles of S-USM were further studied in Figure 3e. They were all found to be up to 150° with a rolling angle of less than 15°, showing excellent underwater repelling and easy flowing away of the oil. Above all, the results showed the superhydrophilicity and underwater superoleophobicity of S-USM, indicating the potential for oil droplets to easily roll away from the S-USM surface without any residue, which is promising for applications separating oil/water mixtures.

### 3.4. The Process of Oil/Water Mixture Separation Using Salt-Containing S-USM

**Figure 4:**
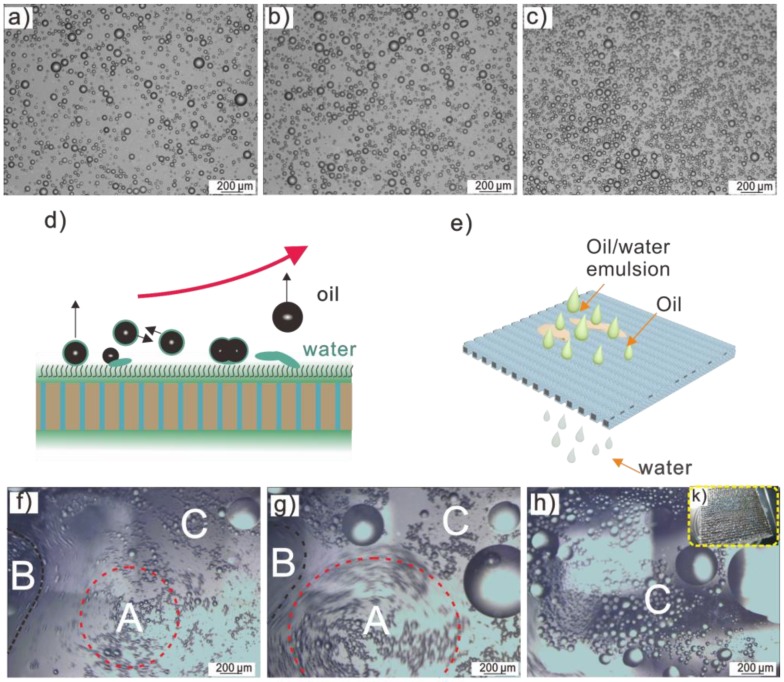
Optical microscopy images of oil-in-water emulsion demulsification and separation. (**a**–**c**) Showing oil demulsification and condensation process of AlCl_3_. (**d**,**e**) A scheme of synchronous demulsification and separation process. (**f**–**h**) Optical microscopy of separation process with water permeating through S-USM, leaving oil droplets above the mesh. (**k**) Optical imagines of milky emulsion on S-USM after separation.

The simulated oil-in-water mixtures were prepared with 10 mL of dodecane, 70 mL of water, and 0.1 g of surfactant, then stirred for 7 h at room temperature. The surfactants were sodium dodecyl sulfate, triethanolamine, Tween 80, and no-surfactant marked as (a), (b), (c), (d) in Figure S4. With the addition of 0.10 g PAM, 0.50 g NaCl, 0.94 g CaCl_2_, 0.92 g AlCl_3_ into 10 mL of the above mixtures, the flocculation and coalescence were enhanced, clarifying the turbid solution. Generally, the presence of electrolytes, such as inorganic salt, influences the surface charge of emulsions by compressing the electrical double layer around the droplet, thus reducing the electrical repulsions and enhancing the gathering of micro oil droplets [45,46]. In detail, the demulsification by AlCl_3_ was observed by optical microscopy at room temperature (RH ~30%) as shown in Figure 4a–c. Compared with the initial emulsion in Figure 4a, an increased number of oil droplets were found above the mixtures in Figure 4b,c, indicating more collisions were happening between the two neighboring emulsions, and that oil droplets aggregated together into larger droplets, until floating up as shown in Figure 4d.

Furthermore, one of the highlights of this work is its use of an in situ demulsification and separation method. On the one hand, demulsification takes place on the nearby surface of salt-containing S-USM. On the other hand, water droplets permeate through the mesh simultaneously, leaving the oil droplets above the mesh (Figure 4e). From Figure 4f,g, oil droplets aggregated and condensed with an increasing area of C, and decreasing area of A and B (A and B represent water fields, C represents oil field). Finally, oil covered the S-USM surface randomly in Figure 4h. As shown in Figure 4k, a milky emulsion was present on S-USM, indicating successful separation.

The surface chemical composition of AlCl_3_-containing S-USM was confirmed by EDS in Figure S5. It is obvious that the elemental content of C (60.02%) and O (34.39%) after separation was more than before separation (with C (58.45%) and O (28.0%)), indicating residual oil on the mesh.

**Figure 5:**
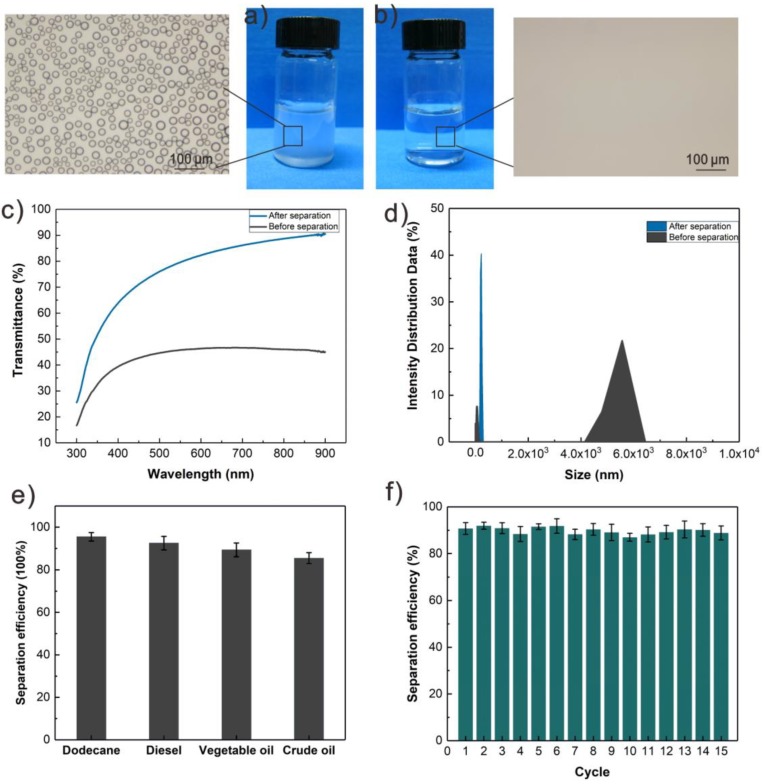
Oil-in-water emulsion separation results. Optical microscopy images of oil-in-water emulsion, (**a**) before and (**b**) after filtration. (**c**,**d**) The ultraviolet transmittance and emulsion size before and after filtration. (**e**) The separation efficiency of dodecane, diesel, vegetable oil, and crude oil. (**f**) Cycle separation test of 15 times of dodecane-in-water mixtures.

To further evaluate the separation ability of AlCl_3_-containing S-USM, a series of mixtures, such as dodecane-in-water, diesel-in-water, vegetable oil-in-water, and crude oil-in-water, was tested. The oil-in-water mixtures were poured onto the S-USM, which was fixed between two glass tubes as shown in Figure S6a,b. Owing to the superhydrophilicity and underwater superoleophobicity of the mesh, water droplets immediately permeated through the mesh by gravity, while the oil droplets stayed above the mesh. As shown in Figure 5a, there was almost no visible oil droplets in the filtrate, while a great amount of oil-in-water emulsions were contained in the as-prepared mixtures before separation (Figure 5b), showing the excellent separation purity of S-USM. In addition, the optical transmittance of filtrate water had an obvious increase compared to the original dodecane-in-water mixture (Figure 5c). Dynamic light scattering (DLS) was conducted to show the decreasing of droplet sizes between origin oil-in-water mixtures and filtrate (Figure 5d).

The separation efficiency was calculated according to

η = (m_1_/m_0_) × 100, (2)

where m_0_ and m_1_ are the mass of the water before separation and filtration, respectively. As shown in Figure 5e, the separation efficiencies for the four kinds of oil-in-water mixtures were all above 80%. The S-USM also has good recycling separation ability. It showed high separation efficiency of dodecane-in-water mixtures, almost 90% in Figure 5f, indicating that the S-USM is applicable for performing repeated rounds of oil/water mixture separation.

The separation flux of filtrate water was calculated by

(3) Flux = Vol/(A·t),

where Vol is filter water volume, A is the area of mesh, and t is the consumed time. As shown in Figure S6c,d, the S-USM had a high initial separation rate, and with extended time, the hydrogel absorbed a high volume of water and swelled, which would decrease the porous area or even blocked the holes.

### 3.5. Special Oil Skimmers Made of S-USM

**Figure 6:**
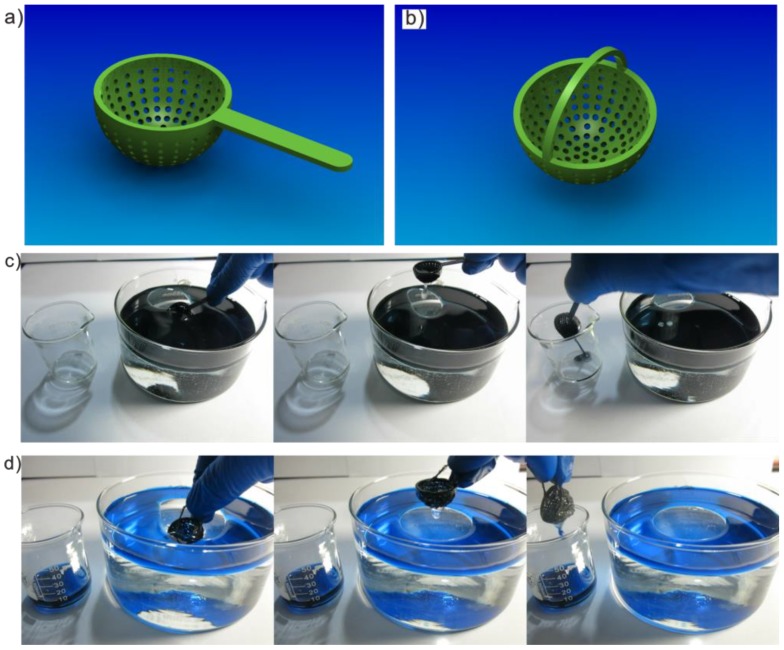
Floating oil removal by 3D-printed spherical oil skimmers. (**a**) The design of a spoon skimmer and (**b**) a barrel skimmer. Collecting floating oil of (**c**) dodecane dyed in blue on water by using spoon skimmer and (**d**) diesel dyed in green with barrel skimmer.

Interestingly, the cooperation of salt further contributed to flocculation and coalescence, and the flocculated oil droplets began to float on water. To better remove floating oil from water, we demonstrated two specific devices useful for separation, called a spoon skimmer and a barrel skimmer, created with the help of 3D printing in fabricating complex structures (Figure 6). In the case of imitating collecting floating oil (dodecane dyed in blue), a spoon skimmer was created (Figure 6a and Figure S7a). The spoon skimmer consisted of two main parts: spherical mesh and a straight hilt which could be convenient for the operator. The design of a spoon skimmer was developed from the S-USM with high separation efficiency of oil-in-water mixtures. Due to more pores in its spherical surface, the spoon skimmer had a faster speed of separation, making it efficient at resolving accidental oil spills, as shown in Figure 6c. Furthermore, a barrel skimmer was successful when applied in another case, to collect floating oil (diesel dyed in green) within deep surroundings. The barrel skimmer was composed of spherical mesh and a curved hilt (Figure 6b and Figure S7b). We demonstrated a collection of floating oil that utilized the barrel skimmer shown in Figure 6d. Both oil skimmers succeeded in removing oil from water, as shown in Figure S8 and Video S1. Combining hydrogel-coated treatment with the new manufacturing technology of 3D printing, a series of specific, useful separation devices can be fabricated, bringing great convenience to personal life.

## 4. Conclusions

In conclusion, we have developed an in situ method to fabricate S-USM and investigated its wettability and separation performance using homemade equipment. The salt-containing S-USM was efficient for a wide range of oil-in-water mixtures, such as dodecane, diesel, vegetable oil, and crude oil, with a separation efficiency of up to 85%. During the recycling separation test, the separation efficiency remained at approximately 90%, showing high repeatability. The mechanism of emulsion destabilization by inorganic salt is often considered to be electrostatic repulsions. In this manner, the salt-containing S-USM could even demulsificate oil/water emulsions, with rapid separation.

Moreover, a key innovation was utilizing the flexible design and fabrication of 3D printing with subsequent hydrogel-coating treatment. The spherical skimmers with hydrogel coatings were facilely created and capable of removing the floating oil. Various oil/water separators can be realized to meet future requirements and bring great convenience to personal life. In view of its simplicity, this work may pave the way for a new method using 3D printing technology, with more practical applications in the fields of separation, hydrogels, electronics, smart robots, and many others.

## Supplementary Materials

The following are available online at https://www.mdpi.com/2073-4360/11/5/774/s1.

## References

[1] Shi Z., Chen X., Wang X., Zhang T., Jin J. (2011). Fabrication of Superstrong Ultrathin Free-Standing Single-Walled Carbon Nanotube Films via a Wet Process. Adv. Funct. Mater..

[2] Chu Z., Feng Y., Seeger S. (2015). Oil/water separation with selective superantiwetting/superwetting surface materials. Angew. Chem..

[3] Chaudhary J.P., Vadodariya N., Nataraj S.K., Meena R. (2015). Chitosan-Based Aerogel Membrane for Robust Oil-in-Water Emulsion Separation. ACS Appl. Mater. Interfaces.

[4] Minas C., Carnelli D., Tervoort E., Studart A.R. (2016). 3D Printing of Emulsions and Foams into Hierarchical Porous Ceramics. Adv. Mater..

[5] Kwon G., Mabry J.M., Kota A.K., Choi W., Tuteja A. (2012). Hygro-responsive membranes for effective oil–water separation. Nat. Commun..

[6] Chen P.-C., Xu Z.-K. (2013). Mineral-Coated Polymer Membranes with Superhydrophilicity and Underwater Superoleophobicity for Effective Oil/Water Separation. Sci. Rep..

[7] Huang B., Li X., Zhang W., Fu C., Wang Y., Fu S. (2019). Study on Demulsification-Flocculation Mechanism of Oil-Water Emulsion in Produced Water from Alkali/Surfactant/Polymer Flooding. Polymers.

[8] Wang C., Yao T., Wu J., Ma C., Fan Z., Wang Z., Cheng Y., Lin Q., Yang B. (2009). Facile Approach in Fabricating Superhydrophobic and Superoleophilic Surface for Water and Oil Mixture Separation. ACS Appl. Mater. Interfaces.

[9] Chakrabarty B., Ghoshal A., Purkait M. (2008). Ultrafiltration of stable oil-in-water emulsion by polysulfone membrane. J. Membr. Sci..

[10] Zhang J., Seeger S. (2011). Polyester Materials with Superwetting Silicone Nanofilaments for Oil/Water Separation and Selective Oil Absorption. Adv. Funct. Mater..

[11] Jin M., Wang J., Yao X., Liao M., Zhao Y., Jiang L. (2011). Underwater Oil Capture by a Three-Dimensional Network Architectured Organosilane Surface. Adv. Mater..

[12] Yu M., Wang Q., Yang W., Xu Y., Zhang M., Deng Q., Liu G. (2019). Facile Fabrication of Magnetic, Durable and Superhydrophobic Cotton for Efficient Oil/Water Separation. Polymers.

[13] Cao J., Zhang J., Zhu Y., Wang S., Wang X., Lv K. (2018). Novel Polymer Material for Efficiently Removing Methylene Blue, Cu(II) and Emulsified Oil Droplets from Water Simultaneously. Polymers.

[14] Zhang Y.-P., Yang J.-H., Li L.-L., Cui C.-X., Li Y., Liu S.-Q., Zhou X.-M., Qu L.-B. (2019). Facile Fabrication of Superhydrophobic Copper-Foam and Electrospinning Polystyrene Fiber for Combinational Oil–Water Separation. Polymers.

[15] Xiong Q., Bai Q., Li C., Lei H., Liu C., Shen Y., Uyama H. (2018). Cost-Effective, Highly Selective and Environmentally Friendly Superhydrophobic Absorbent from Cigarette Filters for Oil Spillage Clean up. Polymers.

[16] Zhang F., Zhang W.B., Shi Z., Wang D., Jin J., Jiang L. (2013). Nanowire-Haired Inorganic Membranes with Superhydrophilicity and Underwater Ultralow Adhesive Superoleophobicity for High-Efficiency Oil/Water Separation. Adv. Mater..

[17] Zhang W., Shi Z., Zhang F., Liu X., Jin J., Jiang L. (2013). Superhydrophobic and Superoleophilic PVDF Membranes for Effective Separation of Water-in-Oil Emulsions with High Flux. Adv. Mater..

[18] Yu Z., Li X., Peng Y., Min X., Yin D., Shao L. (2019). MgAl-Layered-Double-Hydroxide/Sepiolite Composite Membrane for High-Performance Water Treatment Based on Layer-by-Layer Hierarchical Architectures. Polymers.

[19] Chen W., He H., Zhu H., Cheng M., Li Y., Wang S. (2018). Thermo-Responsive Cellulose-Based Material with Switchable Wettability for Controllable Oil/Water Separation. Polymers.

[20] Striemer C.C., Gaborski T.R., McGrath J.L., Fauchet P.M. (2007). Charge- and size-based separation of macromolecules using ultrathin silicon membranes. Nature.

[21] Wang C.-F., Tzeng F.-S., Chen H.-G., Chang C.-J. (2012). Ultraviolet-Durable Superhydrophobic Zinc Oxide-Coated Mesh Films for Surface and Underwater–Oil Capture and Transportation. Langmuir.

[22] Lai Y., Huang J., Cui Z., Ge M., Zhang K.Q., Chen Z., Chi L. (2016). Recent advances in TiO_2_-based nanostructured surfaces with controllable wettability and adhesion. Small.

[23] Zhu M., Xiong R., Huang C. (2019). Bio-based and photocrosslinked electrospun antibacterial nanofibrous membranes for air filtration. Carbohydr. Polym..

[24] Ma W., Zhang M., Liu Z., Kang M., Huang C., Fu G. (2019). Fabrication of highly durable and robust superhydrophobic-superoleophilic nanofibrous membranes based on a fluorine-free system for efficient oil/water separation. J. Membr. Sci..

[25] Ma W., Zhao J., Oderinde O., Han J., Liu Z., Gao B., Xiong R., Zhang Q., Jiang S., Huang C. (2018). Durable superhydrophobic and superoleophilic electrospun nanofibrous membrane for oil-water emulsion separation. J. Colloid Interface Sci..

[26] Ma W., Samal S.K., Liu Z., Xiong R., De Smedt S.C., Bhushan B., Zhang Q., Huang C. (2017). Dual pH- and ammonia-vapor-responsive electrospun nanofibrous membranes for oil-water separations. J. Membr. Sci..

[27] Xue D., Wang Y., Zhang J., Mei D., Wang Y., Chen S. (2018). Projection-Based 3D Printing of Cell Patterning Scaffolds with Multiscale Channels. ACS Appl. Mater. Interfaces.

[28] Yu Y., Yang M., Fu Z., Niu K., Yi C., Hua S., Teng S., Zhao Q. (2016). Fabrication and characterization of electrospinning/3D printing bone tissue engineering scaffold. RSC Adv..

[29] Scholze M., Singh A., Lozano P.F., Ondruschka B., Ramezani M., Werner M., Hammer N. (2018). Utilization of 3D printing technology to facilitate and standardize soft tissue testing. Sci. Rep..

[30] Hardin J.O., Ober T.J., Valentine A.D., Lewis J.A. (2015). Microfluidic Printheads for Multimaterial 3D Printing of Viscoelastic Inks. Adv. Mater..

[31] Ji Q., Zhang J.M., Liu Y., Li X., Lv P., Jin D., Duan H. (2018). A Modular Microfluidic Device via Multimaterial 3D Printing for Emulsion Generation. Sci. Rep..

[32] Yan C., Ji Z., Ma S., Wang X., Zhou F. (2016). 3D Printing as Feasible Platform for On-Site Building Oil-Skimmer for Oil Collection from Spills. Adv. Mater. Interfaces.

[33] Wang X., Cai X., Guo Q., Zhang T., Kobe B., Yang J. (2013). i3DP, a robust 3D printing approach enabling genetic post-printing surface modification. Chem. Commun..

[34] Yang Y., Li X., Zheng X., Chen Z., Zhou Q., Chen Y. (2018). 3d-printed biomimetic super-hydrophobic structure for microdroplet manipulation and oil/water separation. Adv. Mater..

[35] Schneider M., Günter C., Taubert A. (2018). Co-Deposition of a Hydrogel/Calcium Phosphate Hybrid Layer on 3D Printed Poly(Lactic Acid) Scaffolds via Dip Coating: Towards Automated Biomaterials Fabrication. Polymers.

[36] Zhao X., Irvine S.A., Agrawal A., Cao Y., Lim P.Q., Tan S.Y., Venkatraman S.S., Ying T.S., Venkatraman S. (2015). 3D patterned substrates for bioartificial blood vessels—The effect of hydrogels on aligned cells on a biomaterial surface. Acta Biomater..

[37] Raman R., Clay N.E., Sen S., Melhem M., Qin E., Kong H., Bashir R. (2016). 3D printing enables separation of orthogonal functions within a hydrogel particle. Biomed. Microdevices.

[38] Xue Z., Wang S., Lin L., Chen L., Liu M., Feng L., Jiang L. (2011). A Novel Superhydrophilic and Underwater Superoleophobic Hydrogel-Coated Mesh for Oil/Water Separation. Adv. Mater..

[39] Fan J.-B., Song Y., Wang S., Meng J., Yang G., Guo X., Feng L., Jiang L. (2015). Directly Coating Hydrogel on Filter Paper for Effective Oil-Water Separation in Highly Acidic, Alkaline, and Salty Environment. Adv. Funct. Mater..

[40] Wong S., Teng T.-T., Ahmad A.L., Zuhairi A., Najafpour G. (2006). Treatment of pulp and paper mill wastewater by polyacrylamide (PAM) in polymer induced flocculation. J. Hazard. Mater..

[41] Zeng Y., Yang C., Zhang J., Pu W. (2007). Feasibility investigation of oily wastewater treatment by combination of zinc and PAM in coagulation/flocculation. J. Hazard. Mater..

[42] Yakovchenko M., Pinchuk L.G., Kolosova M.M., Filipovich L.A., Masaev V.Y. (2019). Intensification of Wastewater Treatment Processes during Coal Enrichment Using Modified Polyacrylamide Flocculants. IOP Conf. Ser. Earth Environ. Sci..

[43] Ma S., Yan C., Cai M., Yang J., Wang X., Zhou F., Liu W. (2018). Continuous Surface Polymerization via Fe(II)-Mediated Redox Reaction for Thick Hydrogel Coatings on Versatile Substrates. Adv. Mater..

[44] Ma S., Rong M., Lin P., Bao M., Xie J., Wang X., Huck W.T.S., Zhou F., Liu W. (2018). Fabrication of 3D Tubular Hydrogel Materials through On-Site Surface Free Radical Polymerization. Chem. Mater..

[45] Yuan S., Tong M., Wu G. (2011). Destabilization of emulsions by natural minerals. J. Hazard. Mater..

[46] Tong K., Zhang Y., Chu P.K. (2013). Evaluation of calcium chloride for synergistic demulsification of super heavy oil wastewater. Colloids Surfaces A Physicochem. Eng. Asp..

