# Supplementary material for: 3D Printing of an Oil/Water Mixture Separator with In Situ Demulsification and Separation
Source: Polymers (Basel). 2019 May 1;11(5):774. doi: 10.3390/polym11050774 (PMC6571658; doi:10.3390/polym11050774)
Supplement: Supplementary file 1 [file polymers-11-00774-s001.zip › Supporting Information.docx]

**Supplementary Materials**

3D printing of oil/water mixtures separator with in situ demulsification and separation

Changyou Yan ^1,2^, Shuanhong Ma ^1^, Zhongying Ji ^1,2^, Yuxiong Guo^1,3^, Zhilu Liu ^1^, Xiaoqin Zhang ^1,^*, and Xiaolong Wang ^1,3,^*

^1^ State Key Laboratory of Solid Lubrication, Lanzhou Institute of Chemical Physics, Chinese Academy of Sciences, Lanzhou 730000, China;

^2^ Center of Materials Science and Optoelectronics Engineering, University of Chinese Academy of Sciences, Beijing 100039, China;

^3^ Yiwu R&D Centre for Functional Materials, LICP, CAS, Yiwu 322000, China;

***** Correspondence: [zhangxiaoqin@licp.cas.cn](mailto:zhangxiaoqin@licp.cas.cn) (X.Z.); wangxl@licp.cas.cn (X.W.); Tel.: +86-931-4968169


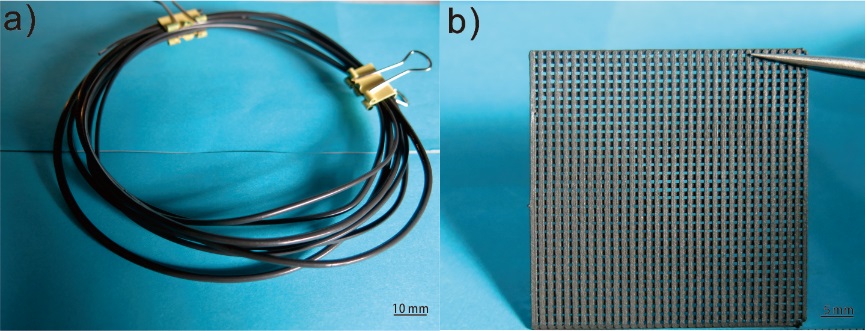


Figure S1. Optical images of a) Fe/PLA composites filament and b) 3D printed mesh. The diameter of Fe/PLA composites filament is close to 1.75 mm.

e

e

e

**
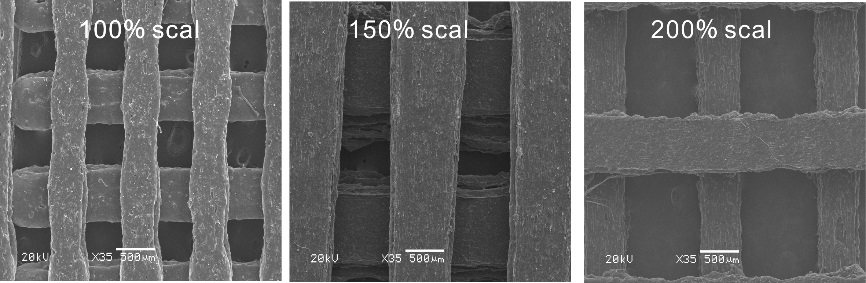
**

Figure S2. The SEM images of printed meshes with different scale.


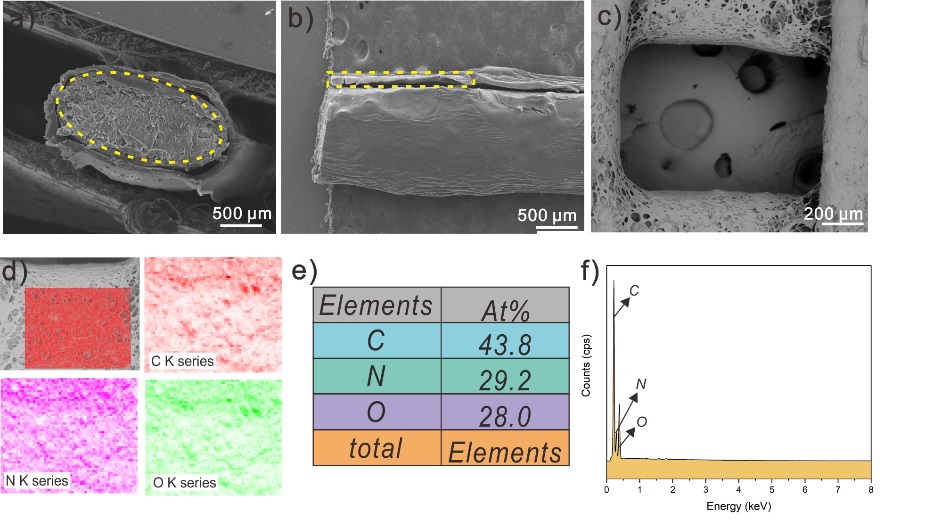


Figure S3. The SEM images of hydrogel layer coated on printed PLA/Fe mesh stick, a) cross section and b) side surface. c) The SEM images of mesh after hydrogel coating. d, e, f) EDS mapping images and element analysis of hydrogel coating.


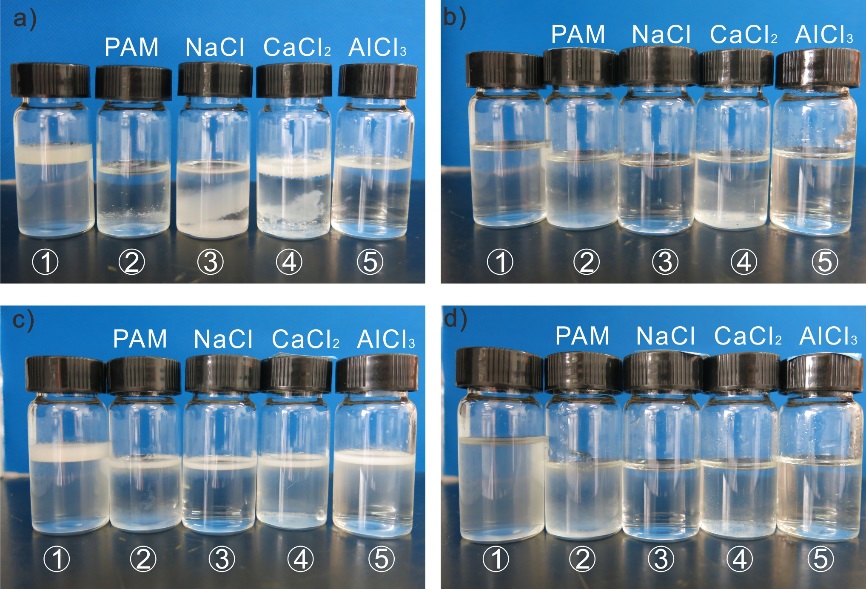


Figure S4. Demulsification effect and optical clarity of dodecane-in-water mixture with the demulsifiers respectively blank, polyacrylamide (PAM), NaCl, CaCl_2_, and AlCl_3_ from left to right. a) surfactant of sodium dodecyl sulfate, b) surfactant of triethanolamine, c) surfactant of tween 80, d) none emulsifier.


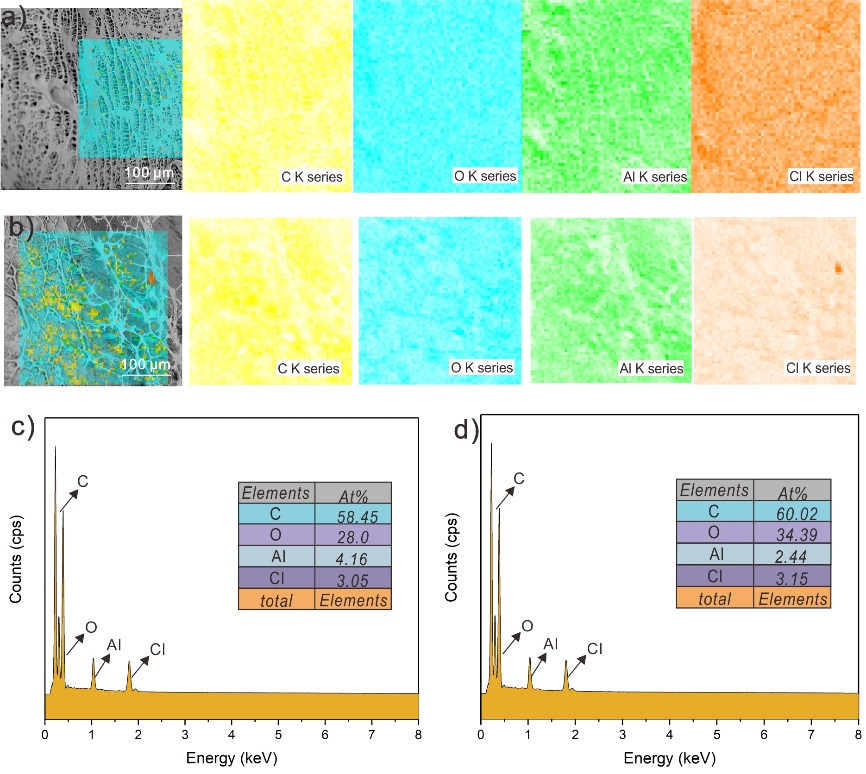


Figure S5. a, b) EDS mapping image of AlCl_3_-contained S-USM before and after oil-in-water emulsion separation. c, d) EDS analysis of the Al element decrease before and after demulsification and separation, respectively.


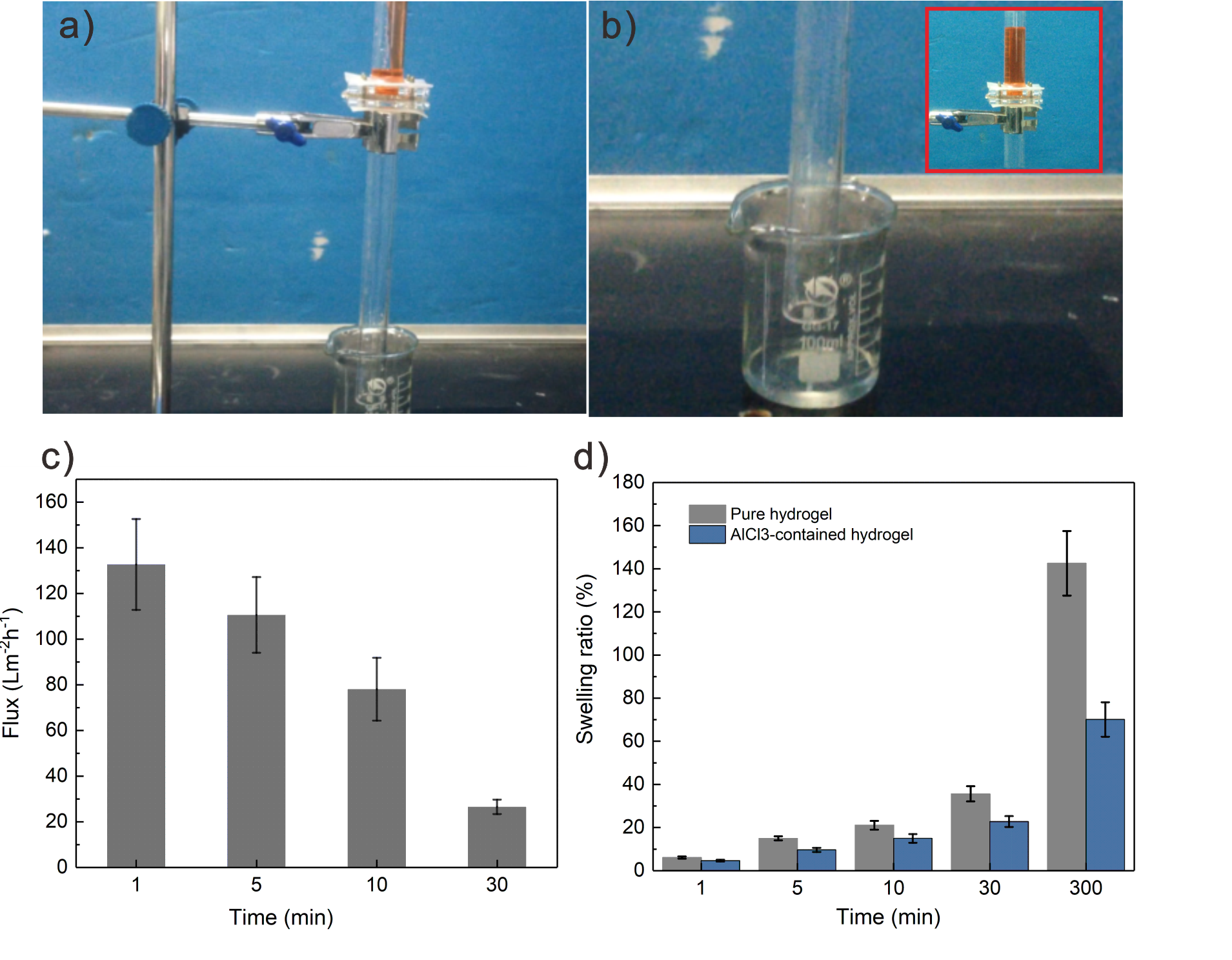


Figure S6. The homemade simple equipment with two glass tubes and sealed by Teflon flanges. a) The oil dyed in red was blocked above the S-USM, b) water permeated through the mesh. The separation of oil/water emulsion was carried out by gravity, c) the flux of dodecane-in-water mixture (5:10, v:v) and d) swelling weight ratio of hydrogel.


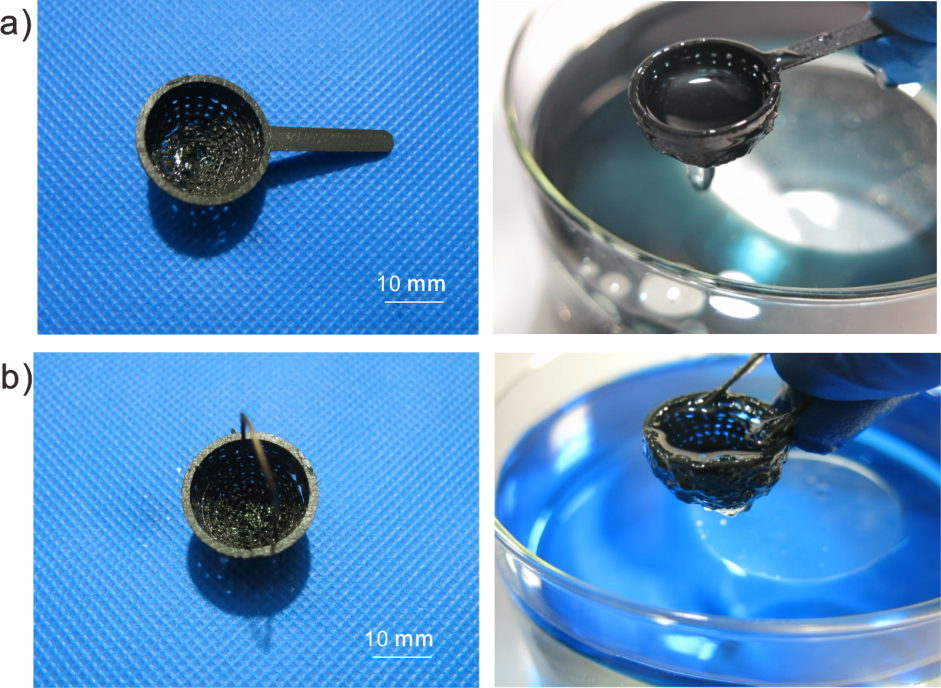


Figure S7. The 3D printed oil-skimmer of a) spherical spoon and b) spherical barrel.


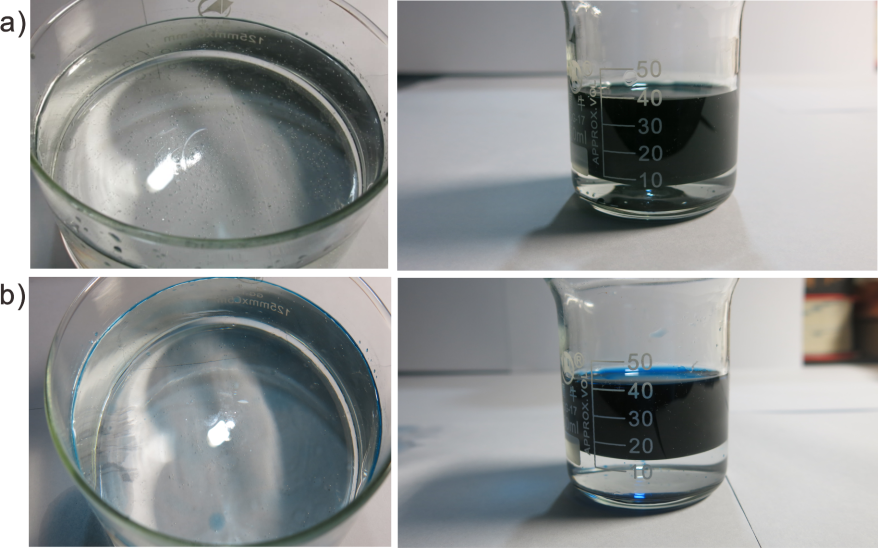


Figure S8. The removal and collected floating oil of a) dodecane dyed in blue and b) diesel dyed in green.

Video S1. The movie of removing floating oil from water a) dodecane dyed in blue and b) diesel dyed in green.
